# Trajectories of Walking Speed and Cause-Specific Mortality: Evidence From England

**DOI:** 10.1093/geroni/igaa057.929

**Published:** 2020-12-16

**Authors:** Giorgio Di Gessa, Paola Zaninotto

**Affiliations:** 1 University College London, London, England, United Kingdom; 2 University College London, London, United Kingdom

## Abstract

Decreased walking speed can predict adverse health-related outcomes such as falls and admissions to hospital. Experiencing fast decline in walking speed has also been associated with increased risk of ‘all-cause’ mortality. In this study, we investigate the links between walking speed trajectories and specific causes of death. We used data from the English Longitudinal Study of Ageing, a large nationally representative survey which collects information biennially on people aged 50 and over in England since 2002. The sample consisted of 4,112 respondents eligible for a walking speed test at baseline who had not died before 2006. Rate of change in walking speed was derived from growth curve models and categorised in three trajectories (slow, moderate, and fast decline). We used competing risk analysis to explore the relationships between these trajectories and mortality, and their interactions with baseline wealth. During a mean of 9.5 years of follow-up, 1543 participants (37%) died (639 from cardiovascula disease -CVD, 311 from respiratory disease -RD, and 593 from cancer). Results suggest a significant difference in mortality across walking speed trajectories (with increased risk of death among those with fast declines) for CVD and RD deaths (P<0.001), even after controlling for baseline characteristics. There was no significant difference for cancer deaths (p=0.44). Further stratified analyses suggested that fast decline was associated with higher CVD and RD mortality even among those with an initial fast walking speed (>1.22 m/s). Strategies to maintain motor performances in later life have the potential to preserve life.

